# A Reduced-Complexity Iterative Bounded Distance Decoder with Random Flipping for Product Codes

**DOI:** 10.3390/e28070824

**Published:** 2026-07-20

**Authors:** Guoming Song, Dongming Pi, Shancheng Zhao

**Affiliations:** College of Information Science and Technology, Jinan University, Guangzhou 510632, China; guomingsong@stu2024.jnu.edu.cn (G.S.); dongmingpi@stu2023.jnu.edu.cn (D.P.)

**Keywords:** product code, soft-aided hard-decision decoding, iterative bounded distance decoding, error-and-erasure decoding, bit flipping

## Abstract

Product codes (PCs) are widely used in high-speed communication systems due to their attractive trade-off between error-correction performance and complexity. To further meet the rapidly growing demand for higher data rates, soft-aided hard-decision decoders (SA-HDDs) have been developed. In this paper, we present a reduced-complexity iterative bounded distance decoder with random flipping (RC-iBDD-RF) for PCs, an SA-HDD that improves the decoding performance while preserving low decoding complexity. RC-iBDD-RF introduces an enhanced reliability metric that incorporates channel log-likelihood ratios (LLRs) and memory from previous iterations to guide random flipping. In addition, an error-and-erasure decoding (EaED) module employing fixed filling patterns, rather than randomly generated ones, is used for post-processing to further reduce the residual error rate. The comparisons for BCH-based PCs show that RC-iBDD-RF achieves both performance gain and complexity reduction with only a slight increase in memory requirement. Moreover, the proposed complexity-storage weighted SNR metric confirms its superior complexity-performance trade-off over iBDD-RF.

## 1. Introduction

Product codes (PCs) and other product-like constructions are widely used in high-speed communication systems due to their attractive trade-off between implementation cost and error-correction performance [1,2]. For systems such as optical transport networks (OTNs), where high throughput and low decoding latency are critical [3], hard-decision decoders remain particularly attractive since they significantly reduce data flow and enable simpler hardware implementations compared with soft-decision decoding schemes [4,5,6].

Extensive research efforts have been devoted to the construction of advanced product-like codes. To improve the performance of classical PCs, staircase codes (SCs) were proposed, in which neighboring blocks are spatially coupled to enhance decoding performance [7]. Hu et al. designed a high-throughput SC encoder achieving an approximate throughput of 119.5 Gbps, demonstrating the feasibility of SCs for real-world OTNs [8]. In [9], an experimental demonstration of the performance advantages of the multi-chain coupled staircase code in a 240-Gbps optical coherent transmission system was presented. In [10], a novel framework termed zipper code was introduced to describe several product-like codes. Within this framework, several high-performance constructions, including multi-chain coupled, Reed–Solomon (RS)-based zipper codes and high-order staircase codes, were proposed [11,12,13,14,15]. These constructions offer increased design flexibility in terms of coupling dimensions, component codes, and decoding schedules, while maintaining compatibility with hard-decision decoding. In addition to product-like code constructions over BCH and RS component codes, recent progress in algebraic coding theory has also explored code constructions and decoding algorithms over richer algebraic structures, such as Gaussian integer rings, Eisenstein integers, quaternion/Hurwitz integers, and octonion integers [16,17,18]. These studies broaden the algebraic foundations of coding theory and may provide useful insights for future code design and decoding algorithms.

However, the low-complexity decoders for product codes remain a critical performance bottleneck. In existing product-like code constructions, Bose–Chaudhuri–Hocquenghem (BCH) or RS codes are commonly employed as component codes [19]. Their low-complexity decoders typically rely on iterative bounded-distance decoding (iBDD), which, however, suffers from inherently limited performance gains [20]. In particular, when the number of errors within a received sequence exceeds the error-correcting capability, a bounded-distance decoder (BDD) may produce miscorrections [21], which can severely degrade the overall decoding performance and even lead to error propagation across iterations. As a result, the error-correction performances of iBDD-based decoders are often significantly inferior to those of the soft-decision decoders, particularly in the moderate-to-high signal-to-noise ratio (SNR) region. To address these limitations while preserving the low complexity of hard-decision decoding, extensive research focuses on the design of soft-aided hard-decision decoders (SA-HDDs).

A variety of SA-HDDs have been proposed in the literature by exploiting soft reliabilities. Log-likelihood ratios (LLRs) were incorporated into the decoding process of iBDD, leading to iBDD with scaled reliability (iBDD-SR) that significantly improves the decoding performance [22]. The parameters of iBDD-SR were optimized in [23]. Building upon iBDD-SR, Liga et al. introduced reliability-aware bit marking into BDD, achieving approximately 0.8 dB performance gain over conventional iBDD [24]. Further advancement was made by Liva et al. through the use of generalized minimum-distance decoding, which substantially reduced the performance gap between traditional iBDD and the Chase–Pyndiah decoder [1], albeit at the cost of increased decoding complexity [25]. Lei et al. proposed an SA-HDD by classifying bits based on their LLRs and selectively flipping those with smaller reliabilities to suppress miscorrections [26]. More recently, refined reliability metrics have been developed in [27]. Other low-complexity SA-HDDs can be found in [28,29]. To further improve decoding performance, iterative bounded-distance decoding with random flipping (iBDD-RF) [30] was proposed, resulting in a remarkable performance gain.

However, a significant performance gap remains between existing HDDs and the Chase–Pyndiah decoder [1] under comparable complexity constraints. In this paper, we propose a reduced-complexity iterative bounded-distance decoder with random flipping (RC-iBDD-RF), a soft-aided binary message-passing decoding scheme with refined bit reliability to enable low-cost random flipping. Specifically, RC-iBDD-RF augments the conventional weighted combination of the BDD output and channel LLR with a memory term that retains useful information from previous iterations. Specifically, unlike the existing iBDD-RF [30] that relies mainly on the current BDD output and channel LLR, RC-iBDD-RF augments its conventional weighted combination with a memory term to retain useful information from previous iterations. This enhanced reliability reduces erroneous flips and improves the overall error-correction capability.

The main contributions of this paper are summarized as follows. First, we develop an enhanced reliability metric that incorporates past decoding states into the soft reliability of each bit, enabling more informed bit-flipping decisions. Second, we incorporate the error-and-erasure decoder (EaED) with fixed filling patterns as a post-processing module to reduce the residual errors. Third, we provide a detailed analysis of the memory and computational complexities of the proposed decoder. Finally, simulation results demonstrate that RC-iBDD-RF reduces the decoding complexity while achieving performance gains.

## 2. Preliminaries

### 2.1. Product Codes

Product codes are a class of algebraic block codes constructed by the Cartesian product of two or more shorter component codes. Since their introduction by Elias in 1954 [31], product codes have been extensively investigated and have found widespread use in high-speed communication systems due to their favorable trade-off between decoding complexity and error-correction performance.

A PC can be represented by an $n_{2}\times n_{1}$ matrix with $n_{2}$ rows and $n_{1}$ columns, where each row and each column corresponds to a codeword of a binary linear block code. Specifically, the row component code is denoted by $C_{1}\left[n_{1},k_{1},t_{1}\right]$, while the column component code is denoted by $C_{2}\left[n_{2},k_{2},t_{2}\right]$. Here, $n_{1}$ and $n_{2}$ denote the code lengths, $k_{1}$ and $k_{2}$ denote information dimensions, and $t_{1}$ and $t_{2}$ denote the error-correcting capabilities of the binary linear block codes $C_{1}$ and $C_{2}$, respectively.

The structure of a PC consists of four sub-matrices: an information-bit matrix of size $k_{1}\times k_{2}$; a row-parity matrix of size $\left(n_{1}-k_{1}\right)\times k_{2}$; a column-parity matrix of size $\left(n_{2}-k_{2}\right)\times k_{1}$; and a parity-of-parity matrix of size $\left(n_{1}-k_{1}\right)\times \left(n_{2}-k_{2}\right)$. Accordingly, the code rate of the PC can be expressed as

(1) $$R=R_{1}\cdot R_{2}=\frac{k_{1}k_{2}}{n_{1}n_{2}},$$

where $R_{1}$ and $R_{2}$ denote the code rates of the component codes $C_{1}$ and $C_{2}$, respectively. When both rows and columns employ the same binary linear block code C[n,k,t], the code rate of the resulting PC is

(2) $$R=\frac{k^{2}}{n^{2}}.$$

### 2.2. The Conventional iBDD-RF

**Figure 1:**
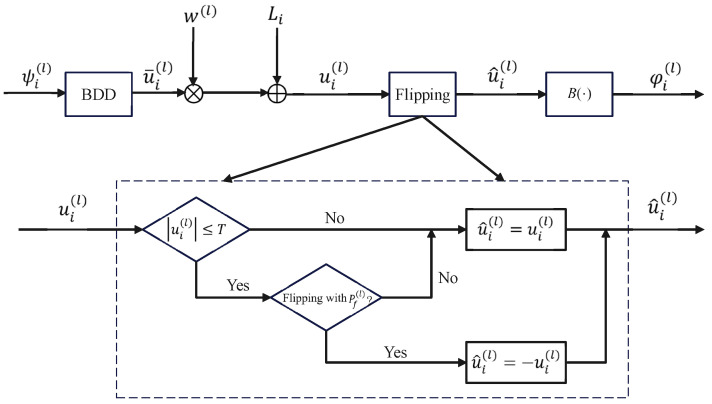
The decoding scheme of iBDD-RF.

The iBDD-RF is a novel binary message-passing decoder that randomly flips bits with low reliability [30]. For completeness, we present in Figure 1 the block diagram of iBDD-RF. We use $\psi^{\left(l\right)}=\left(\psi_{1}^{\left(l\right)},\cdots ,\psi_{n}^{\left(l\right)}\right)$ and $\varphi^{\left(l\right)}=\left(\varphi_{1}^{\left(l\right)},\cdots ,\varphi_{n}^{\left(l\right)}\right)$ to represent the input sequence and output sequence of a row or a column of the iBDD-RF in the *l*-th iteration, respectively.

Given the input $\psi^{\left(l\right)}=\left(\psi_{1}^{\left(l\right)},\cdots ,\psi_{n}^{\left(l\right)}\right)$, let ${\bar{c}}^{\left(l\right)}=\left({\bar{c}}_{1}^{\left(l\right)},\cdots ,{\bar{c}}_{n}^{\left(l\right)}\right)$ denote the decoding output of the BDD at the *l*-th iteration. If the BDD declares a success, we have ${\bar{u}}_{i}^{\left(l\right)}={\left(-1\right)}^{{\bar{c}}_{i}^{\left(l\right)}}$. Otherwise, we have ${\bar{u}}_{i}^{\left(l\right)}=0$. Similar to iBDD-SR, the soft reliability $u_{i}^{\left(l\right)}$ of the *i*-th code bit $c_{i}$ is given as

(3) $$u_{i}^{\left(l\right)}=w^{\left(l\right)}{\bar{u}}_{i}^{\left(l\right)}+L_{i},$$

where $w^{\left(l\right)}$ is the scaling factor at the *l*-th iteration, and $L_{i}$ is the channel LLR of the *i*-th code bit. Let *T* be a given threshold. If $|u_{i}^{\left(l\right)}|<T$, $u_{i}^{\left(l\right)}$ is flipped with a probability of $P_{f}^{\left(l\right)}$. The output after flipping is denoted as ${\hat{u}}_{i}^{\left(l\right)}$. That is, we have

(4) $${\hat{u}}_{i}^{\left(l\right)}=\left\{\begin{array}{cc} u_{i}^{\left(l\right)} & \mathrm{if}\;\left|u_{i}^{\left(l\right)}\right|>T\\ u_{i}^{\left(l\right)} & \mathrm{if}\;\left|u_{i}^{\left(l\right)}\right|\leq T,\mathrm{with}\;\mathrm{probability}\;1-P_{f}^{\left(l\right)}\\ -u_{i}^{\left(l\right)} & \mathrm{if}\;\left|u_{i}^{\left(l\right)}\right|\leq T,\mathrm{with}\;\mathrm{probability}\;P_{f}^{\left(l\right)}. \end{array}\right.$$

Finally, ${\hat{u}}^{\left(l\right)}=\left({\hat{u}}_{1}^{\left(l\right)},{\hat{u}}_{2}^{\left(l\right)},\cdots ,{\hat{u}}_{n}^{\left(l\right)}\right)$ is taken as the input to the hard-decision operator B(·) to obtain the output $\varphi^{\left(l\right)}$ as $\varphi^{\left(l\right)}=B\left({\hat{u}}^{\left(l\right)}\right)=\left(B\left({\hat{u}}_{1}^{\left(l\right)}\right),B\left({\hat{u}}_{2}^{\left(l\right)}\right),\cdots ,B\left({\hat{u}}_{n}^{\left(l\right)}\right)\right)$, where

(5) $$B\left(x\right)=\left\{\begin{array}{cc} 0, & x>0\\ 1, & x\leq 0. \end{array}\right.$$

### 2.3. Error and Erasure Decoder

The EaED performs two BDDs, denoted by $D_{1}$ and $D_{2}$, and then selects the more reliable result as the final output. The input sequence to EaED is given by

(6) $$y\in {\left\{0,\;?,\;1\right\}}^{n},$$

where *n* denotes the code length, the symbol “?” denotes an erased bit. We assume that the number of erased bits in the input sequence is *E*, and that the minimum Hamming distance of the component code is $d_{\min}$. The output sequence of EaED is denoted by w.

If $E\geq d_{\min}$, EaED does not perform decoding and keeps the input sequence unchanged. We then have w=y.

If $E<d_{\min}$, two new input sequences $y^{\left(1\right)}$ and $y^{\left(2\right)}$ are constructed from y and fed into $D_{1}$ and $D_{2}$, respectively. The constructions of $y^{\left(1\right)}$ and $y^{\left(2\right)}$ are described in the following. For the non-erased positions, the two sequences are identical to the input sequence. For the erased positions, two filling patterns, $p^{\left(1\right)}$ and $p^{\left(2\right)}$, are used to replace the erased bits, where $p^{\left(1\right)}$ and $p^{\left(2\right)}$ are complementary to each other. In the iterative decoder of [32], the filling patterns $p^{\left(1\right)}$ and $p^{\left(2\right)}$ are randomly generated for each execution of EaED. The decoding output of EaED is given below:

- If both $D_{1}$ and $D_{2}$ fail to decode, the input sequence is retained, and we have w=y.
- If one of the two decoders succeeds while the other fails, the decoding result of the successful decoder is selected as the output w.
- If both $D_{1}$ and $D_{2}$ decode successfully, the output is determined as follows. Let $r^{\left(1\right)}$ and $r^{\left(2\right)}$ denote the two decoding outputs. The Hamming distances of $r^{\left(1\right)}$ and $r^{\left(2\right)}$ to y are denoted by $\delta_{1}$ and $\delta_{2}$, respectively. Based on $\delta_{1}$ and $\delta_{2}$, the output w is given by
  (7) $$w=\left\{\begin{array}{cc} r^{\left(1\right)}, & \mathrm{if}\;\delta_{1}<\delta_{2}\\ r^{\left(2\right)}, & \mathrm{if}\;\delta_{1}>\delta_{2}\\ r^{\left(1\right)}\;\mathrm{or}\;r^{\left(2\right)}, & \mathrm{if}\;\delta_{1}=\delta_{2}. \end{array}\right.$$

## 3. The Proposed Reduced-Complexity Iterative Bounded Distance Decoder with Random Flipping

In this paper, we propose the following binary message passing decoder, which is a modification of iBDD-RF [30]. For ease of presentation, the number of iterations is indexed according to the number of half-iterations, i.e., the row decoding and column decoding at the *l*-th iteration are represented as the (2l−1)-th half-iteration and the 2l-th half-iteration.

### 3.1. Enhanced Soft Reliability

**Figure 2:**
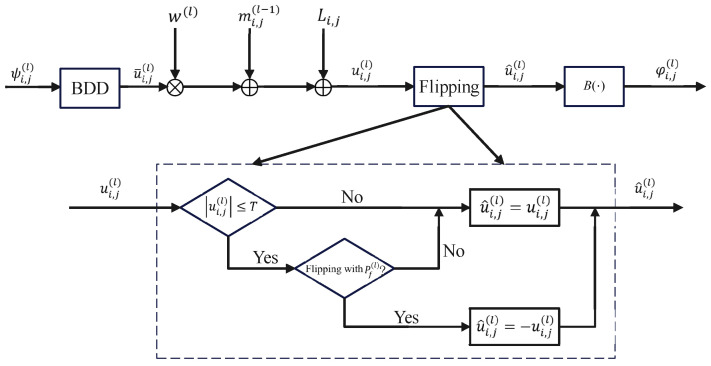
The decoding scheme of RC-iBDD-RF.

We use the notations of iBDD-RF [30] in this paper to present the proposed reduced-complexity iBDD-RF (RC-iBDD-RF). For completeness, we present the block diagram of RC-iBDD-RF in Figure 2. Let $\varphi_{i}^{\left(l\right)}=\left(\varphi_{i,1}^{\left(l\right)},\cdots ,\varphi_{i,n}^{\left(l\right)}\right)$ denote the output sequence of the *i*-th row at the *l*-th half-iteration. For the *l*-th half-iteration, the input of BDD of the *i*-th row is $\psi_{i}^{\left(l\right)}=\left(\psi_{i,1}^{\left(l\right)},\cdots ,\psi_{i,n}^{\left(l\right)}\right)$, and the decoding output of BDD is ${\bar{c}}_{i}=\left({\bar{c}}_{i,1},\cdots ,{\bar{c}}_{i,n}\right)$. In RC-iBDD-RF, the decoding reliability ${\bar{u}}_{i,j}^{\left(l\right)}$ of the BDD for the *j*-th position of the *i*-th row at the *l*-th half-iteration is given as

(8) $${\bar{u}}_{i,j}^{\left(l\right)}=\left\{\begin{array}{cc} 0 & \mathrm{if}\;\mathrm{BDD}\;\mathrm{fails}\\ {\left(-1\right)}^{{\bar{c}}_{i,j}} & \mathrm{if}\;\mathrm{BDD}\;\mathrm{succeeds}. \end{array}\right.$$

In iBDD-RF, the soft reliability $u_{i,j}^{\left(l\right)}$ is computed as $u_{i,j}^{\left(l\right)}=w^{\left(l\right)}{\bar{u}}_{i,j}^{\left(l\right)}+L_{i,j}$, where $w^{\left(l\right)}$ is the scaling factor, and $L_{i,j}$ is the channel LLR. It can be observed that, in iBDD-RF, the soft reliability $u_{i,j}^{\left(l\right)}$ does not account for either the number of bits corrected by BDD or the outcomes of the most recent decoding half-iteration. First, the probability of miscorrection in BDD increases with the number of corrected bits. Second, once a decoding success is declared in the most recent decoding half-iteration, the corresponding decision should not be easily reversed. These observations motivate the following enhanced soft reliability metric.

Let $s_{i}^{\left(l-1\right)}$ denote the status of the *i*-th BDD decoder at the (l−1)-th half-iteration. We have

(9) $$s_{i}^{\left(l-1\right)}=\left\{\begin{array}{cc} 0 & \mathrm{if}\;\mathrm{BDD}\;\mathrm{fails}\\ t+1-\delta & \mathrm{if}\;\mathrm{BDD}\;\mathrm{succeeds}, \end{array}\right.$$

where *t* is the error correcting capability of the component code, and δ denotes the number of bits corrected by BDD. Accordingly, we define the *historical reliability* $m_{i,j}^{\left(l-1\right)}$ of the (i,j) bit at the (l−1)-th half-iteration as

(10) $$m_{i,j}^{\left(l-1\right)}=\beta^{\left(l-1\right)}{\left(-1\right)}^{\varphi_{i,j}^{\left(l-1\right)}}s_{j}^{\left(l-1\right)},$$

when *l* is even, and as

(11) $$m_{i,j}^{\left(l-1\right)}=\beta^{\left(l-1\right)}{\left(-1\right)}^{\varphi_{i,j}^{\left(l-1\right)}}s_{i}^{\left(l-1\right)},$$

when *l* is odd. Here, $\beta^{\left(l\right)}$ is a given scaling factor, $\varphi_{i,j}^{\left(l-1\right)}$ is the output of last half-iteration. The soft reliability $u_{i,j}^{\left(l\right)}$ in RC-iBDD-RF is given as

(12) $$u_{i,j}^{\left(l\right)}=w^{\left(l\right)}{\bar{u}}_{i,j}^{\left(l\right)}+m_{i,j}^{\left(l-1\right)}+L_{i,j}.$$

Given the soft reliability $u_{i,j}^{\left(l\right)}$, we attempt to flip bits randomly. Let *T* be a given threshold. If $|u_{i,j}^{\left(l\right)}|<T$, $u_{i,j}^{\left(l\right)}$ is flipped with a probability of $P_{f}^{\left(l\right)}$. The output result after flipping is denoted as ${\hat{u}}_{i,j}^{\left(l\right)}$. That is, we have

(13) $${\hat{u}}_{i,j}^{\left(l\right)}=\left\{\begin{array}{cc} u_{i,j}^{\left(l\right)} & \mathrm{if}\;\left|u_{i,j}^{\left(l\right)}\right|>T\\ u_{i,j}^{\left(l\right)} & \mathrm{if}\;\left|u_{i,j}^{\left(l\right)}\right|\leq T,\mathrm{with}\;\mathrm{probability}\;1-P_{f}^{\left(l\right)}\\ -u_{i,j}^{\left(l\right)} & \mathrm{if}\;\left|u_{i,j}^{\left(l\right)}\right|\leq T,\mathrm{with}\;\mathrm{probability}\;P_{f}^{\left(l\right)}. \end{array}\right.$$

Finally, ${\hat{u}}_{i}^{\left(l\right)}$ is taken as the input to the hard-decision operator B(·). The output $\varphi_{i}^{\left(l\right)}$ of the RC-iBDD-RF at the *l*-th half-iteration is $\varphi_{i}^{\left(l\right)}=B\left({\hat{u}}_{i}^{\left(l\right)}\right)=\left(B\left({\hat{u}}_{i,1}^{\left(l\right)}\right),B\left({\hat{u}}_{i,2}^{\left(l\right)}\right),\cdots ,B\left({\hat{u}}_{i,n}^{\left(l\right)}\right)\right)$. For completeness, Algorithm 1 presents the procedure of a half-iteration of RC-iBDD-RF.

**Algorithm 1:** The Decoding Procedure of a Half-Iteration of RC-iBDD-RF

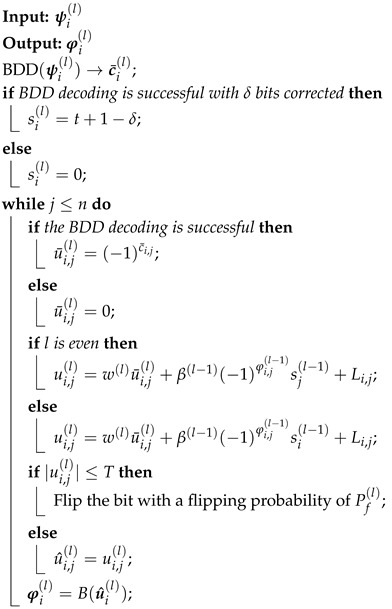



Remark: Unlike conventional SA-iBDD and reliability-combining methods that mainly rely on channel reliability $L_{i,j}$ or current decoding output $\varphi_{i,j}^{\left(l\right)}$, the proposed metric further incorporates the past decoding information $m_{i,j}^{\left(l-1\right)}$ of each bit. Hence, bits with similar channel reliabilities can be assigned different flipping priorities according to their decoding histories. This memory-aided design allows RC-iBDD-RF to focus random flipping on persistently unreliable bits.

### 3.2. Post-Processing by EaED

However, similar to iBDD-RF, the decoding output of RC-iBDD-RF is not guaranteed to be a codeword, which may lead to a high error floor. To further improve the performance of RC-iBDD-RF, we propose to replace the last two half-iterations of BDD with EaED, where the enhanced reliability is used as the erasure criterion. First, to effectively control the decoding complexity, we apply erasure for EaED only once, i.e., at the penultimate half-iteration of RC-iBDD-RF. Second, in the proposed decoder, the two filling patterns $p^{\left(1\right)}$ and $p^{\left(2\right)}$ in EaED are fixed as the all-zero vector 0 and the all-one vector 1.

The details of the last two half-iterations with EaED are given as follows:

- Bits whose absolute reliabilities $|u_{i,j}^{\left(l\right)}|$ are smaller than a predefined threshold $T_{\mathrm{era}}$ are erased.
- If an erased bit is successfully recovered during the penultimate half-iteration, then in the subsequent half-iteration, this bit is no longer in the erased state.
- If an erased bit is not recovered during the penultimate half-iteration, it remains in the erased state in the subsequent half-iteration.

In [32], the two filling patterns are randomly generated for each execution of EaED, which incurs a higher complexity than the proposed scheme. We chose these two fixed filling patterns since erasing is executed only once in the proposed decoder.

In the following, we analyze the error-correcting capability of the ideal EaED (iEaED) with fixed filling patterns 0 and 1 using a genie-aided method. Since miscorrection is not allowed in iEaED, its decoding rule can be expressed as

(14) $$\mathrm{iEaED}\left(y\right)=\left\{\begin{array}{cc} x & \mathrm{if}\;\mathrm{EaED}(y)=x\\ y & \mathrm{otherwise}. \end{array}\right.$$

where x denotes the transmitted codeword. Although such an ideal decoder is not implementable in practice, anchor decoders enable the decoding performance to approach that of an ideal miscorrection-free decoder. Moreover, the miscorrection-free assumption enables a quantitative mathematical analysis of the probability that EaED successfully decodes a component code.

Following [32], let $P_{s}\left(D,E\right)$ denote the probability that iEaED successfully decodes when *E* bits are erased and *D* bits are erroneous. Let *t* denote the error-correcting capability of the component code and *e* denote the number of positions in $p^{\left(1\right)}=0$ that differ from the transmitted codeword x. Following [32], the decoding success probability of iEaED with fixed filling patterns is given by

(15) Ps(D,E)=12D+E<dmin0E≥dminorD>t21−E∑e=0t−DEeotherwise.

It can be seen that when $2D+E<d_{\min}$, EaED always decodes successfully. If all erroneous bits are erased, the EaED can correct up to $d_{\min}-1$ errors, which is significantly higher than the error-correction capability *t* of BDD.

The above analysis shows the potential advantages of EaED in error correction. Hence, we propose to incorporate EaED into RC-iBDD-RF to obtain additional performance gain. Specifically, EaED is employed in the last two half-iterations of RC-iBDD-RF as a post-processing procedure.

## 4. The Analysis of Complexity

In [30], a detailed complexity comparison showed the advantage of iBDD-RF in terms of computational complexity when compared with existing SA-HDDs. Therefore, in the following, we mainly focus on comparing the complexity of RC-iBDD-RF with that of iBDD-RF. We consider memory storage and computational complexity in this paper and briefly discuss the practical implementation implications at the end of this section.

### 4.1. Memory Storage Analysis

For RC-iBDD-RF, it is required to store $w^{\left(l\right)}$, $\beta^{\left(l\right)}$, the probability of flipping $p_{f}^{\left(l\right)}$, decoding status $s^{\left(l\right)}$, and the channel LLR. We consider a BCH component code C[n,k,t] with *q*-bit quantization. Let $l_{\mathrm{RC}}$ denote the number of iterations for the proposed RC-iBDD-RF. The number of decoding iterations is set as $l_{\mathrm{RC}}=l_{\mathrm{RF}}=l_{\mathrm{iBDD}}=12$, following the common practice for HDD-based product-code decoders. This setting is also verified by our preliminary convergence tests, which show that further iterations provide only negligible performance improvement while increasing decoding complexity.

- For a PC, a complete iteration consists of a row half-iteration and a column half-iteration. Therefore, the memory required to store $w^{\left(l\right)}$, $\beta^{\left(l\right)}$, and $p_{f}^{\left(l\right)}$ is $2ql_{\mathrm{RF}}$.
- The maximum possible number of statuses is t+1. Since the error correction capability *t* of the BCH component code is typically smaller than four, three bits are enough for storing the status of a row or a column. As a result, the total memory requirement of the statuses is 3n.
- For the channel LLR, its memory requirement is $n^{2}q$.

Thus, the additional memory requirement of RC-iBDD-RF, when compared with iBDD, is

(16) $$M_{\mathrm{RC}\text{-}\mathrm{iBDD}\text{-}\mathrm{RF}}=q\left(6l_{\mathrm{RC}}+n^{2}\right)+3n.$$

It was shown in [30] that the additional memory requirement of iBDD-RF, when compared with iBDD, is $M_{\mathrm{iBDD}\text{-}\mathrm{RF}}=q\left(4l_{\mathrm{RF}}+n^{2}\right)$.

- For $C_{1}\left[255,231,3\right]$, n=255, $l_{\mathrm{RC}}=l_{\mathrm{RF}}=12$, the memory requirement of RC-iBDD-RF is calculated as 65,097q+765 bits, while that of iBDD-RF is 65,073q bits, where *q* is the quantization bit width of the channel LLR. Therefore, the extra memory of RC-iBDD-RF over iBDD-RF is only 24q+765 bits.
- For $C_{2}\left[256,239,2\right]$, n=256, $l_{\mathrm{RC}}=l_{\mathrm{RF}}=12$, the corresponding memory requirements are 65,608q+768 bits and 65,584q bits, respectively, and the extra memory is 24q+768 bits.

In practical implementations, the quantization bit width *q* is typically no more than 10 bits. These results show that the additional memory requirement of RC-iBDD-RF is limited.

### 4.2. Computational Complexity Analysis

In this subsection, we analyze the computational complexity of RC-iBDD-RF. Similar to iBDD-RF, the main computational complexity of RC-iBDD-RF arises from the execution of the Berlekamp–Massey (BM) algorithm in BDD and the generation of random numbers.

**Figure 3:**
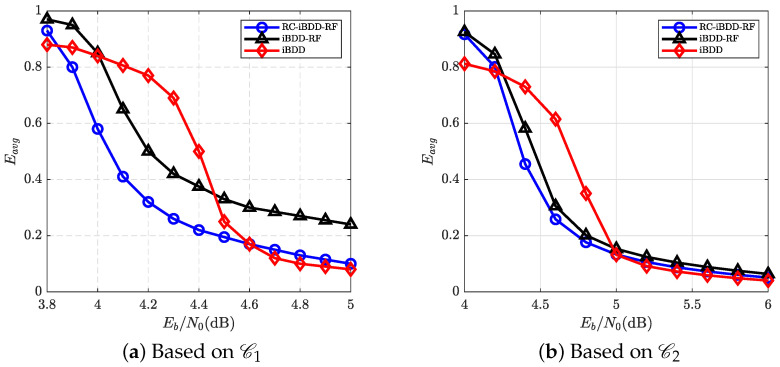
The average execution numbers $E_{\mathrm{avg}}$ of BDD for RC-iBDD-RF and iBDD-RF.

The computational complexity of a single execution of the BM algorithm is $O(n^{2})$. Let $E_{\mathrm{avg}}$ denote the average execution number of the BM algorithm per codeword in a single decoding iteration, which is defined as

(17) $$E_{\mathrm{avg}}=\frac{E_{\mathrm{total}}}{2l_{\mathrm{RC}}n},$$

where $E_{\mathrm{total}}$ is the total execution number of BDD during decoding. Since each iteration involves decoding all codewords in both row and column directions, the overall computational complexity of the BM algorithm in RC-iBDD-RF can be written as $O\;\left(2l_{\mathrm{RC}}E_{\mathrm{avg}}n^{3}\right)$. It can be observed that this expression is consistent with that of iBDD-RF. Hence, to compare the computational complexities of RC-iBDD-RF and iBDD-RF, we only need to compare their $E_{\mathrm{avg}}$. Figure 3a,b, we present the values of $E_{\mathrm{avg}}$ for the RC-iBDD-RF. We select the BCH codes $C_{1}\left[255,231,3\right]$ and $C_{2}\left[256,239,2\right]$ as the component codes. The average execution numbers of the BM algorithm of iBDD-RF are also given in Figure 3a,b. Based on these two figures, we can make the following observations:

- The average execution number of BM in RC-iBDD-RF is lower than that of iBDD-RF.
- The average execution number of BM in RC-iBDD-RF is comparable to that of iBDD.

**Figure 4:**
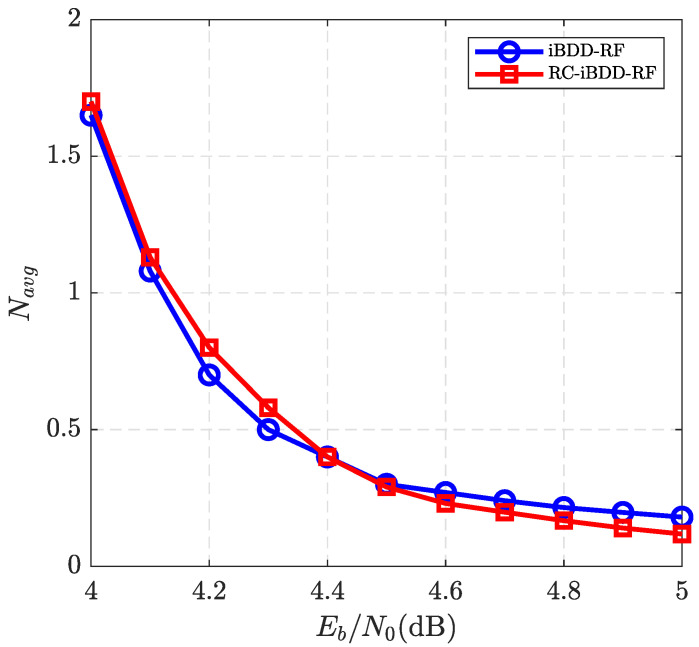
The $N_{\mathrm{avg}}$ for RC-iBDD-RF and iBDD-RF, where $C_{1}$ is selected as the component code.

We then consider the complexity of random number generation in RC-iBDD-RF. Since the filling patterns $p^{\left(1\right)}$ and $p^{\left(2\right)}$ are chosen as the all-zero and all-one sequences, random number generation in RC-iBDD-RF is only required for bit flipping. We use $N_{\mathrm{avg}}$ to denote the average number of random numbers required for each codeword in a single iteration. Thus, the computational complexity associated with random number generation in RC-iBDD-RF is $O(2l_{\mathrm{RC}}nN_{\mathrm{avg}})$. In Figure 4, we present the values of $N_{\mathrm{avg}}$ for RC-iBDD-RF and iBDD-RF, where the code $C_{1}$ is selected as the component code. It can be seen that the two decoders admit comparable average numbers for random number generation. Hence, the complexities of generating random numbers in RC-iBDD-RF and iBDD-RF are comparable.

### 4.3. Complexity Comparison

In this subsection, we provide a detailed comparison of the additional memory requirements relative to iBDD and the computational complexities of RC-iBDD-RF, iBDD-RF, and iBDD. For clarity, Table 1 summarizes their additional memory overheads and the corresponding computational complexities, where $l_{\mathrm{iBDD}}$ denotes the number of iterations for iBDD.

In the following, we illustrate the complexity advantage of RC-iBDD-RF through a practical example. The code $C_{1}$ is selected as the component code. We consider the SNR $E_{b}/N_{0}=5.0$ dB. The number of decoding iterations is set as $l_{\mathrm{RC}}=l_{\mathrm{RF}}=l_{\mathrm{iBDD}}=12$. Similar to [30], BDD is performed at the last two iterations of iBDD-RF.

- From Figure 3a and Figure 4, we have $E_{\mathrm{avg}}=0.105$ and $N_{\mathrm{avg}}=0.155$. Substituting these values into the expression in Table 1, the computational complexity of RC-iBDD-RF can be estimated as $O\left(2.52n^{3}\right)+O\left(3.72n\right)$.
- From Figure 3a and Figure 4, we have $E_{\mathrm{avg}}=0.243$ and $N_{\mathrm{avg}}=0.193$. Substituting these values into the expression in Table 1, the computational complexity of iBDD-RF can be estimated as $O\left(5.832n^{3}\right)+O\left(3.86n\right)$.
- From Figure 3a, we have $E_{\mathrm{avg}}=0.081$. Substituting this value into the expressions in Table 1, the computational complexity of iBDD can be estimated as $O(1.944n^{3})$.

It can be seen from this example that the proposed algorithm achieves a 56.79% reduction in computational complexity when compared with iBDD-RF. Compared with iBDD, the complexity of RC-iBDD-RF increases by about 29.63%. From an implementation perspective, RC-iBDD-RF still preserves the standard row-column BDD decoding structure of iBDD-based decoders. Therefore, the main BDD modules can be reused. Meanwhile, the extra memory access is mainly related to the quantized channel LLRs and the stored decoding states, which can be scheduled together with the row and column decoding process. Moreover, since the EaED module uses fixed filling patterns and is applied only once as post-processing, it does not introduce additional global decoding iterations. Hence, RC-iBDD-RF maintains an implementation-friendly structure for high-speed communication systems.

## 5. Performance Comparison

Extensive simulation results are presented to demonstrate the performance advantages of RC-iBDD-RF.

### 5.1. Parameter Optimization

The scaling factors and the flipping probability $p_{f}^{\left(l\right)}$ in RC-iBDD-RF are all related to the number of iterations, which makes the parameter optimization of RC-iBDD-RF complex. However, no suitable theoretical method is currently available for parameter optimization. Therefore, following the dynamic reliability score decoder (DRSD) [32] and iBDD-RF [30], we employ the hyperparameter optimization tool *optuna* [33] to optimize the parameters of RC-iBDD-RF.

The parameters of RC-iBDD-RF include $w^{\left(l\right)},\beta^{\left(l\right)},p_{f}^{\left(l\right)},T$, and $T_{\mathrm{era}}$. We set the objective function as the required $E_{b}/N_{0}$ at a target bit error rate (BER) of $10^{-3}$. Specifically, the parameter set suggested by *Optuna* is used as the input, and the corresponding $E_{b}/N_{0}$ achieving the target BER is obtained as the output. This value is then fed back to *Optuna*, which iteratively samples and searches for the optimal hyperparameter combination based on previous results.

In this paper, we set the maximum number of decoding iterations to $l_{\max}=12$. The search ranges of the optimized parameters are $w^{\left(l\right)}\in \left[3,10\right]$, $\beta^{\left(l\right)}\in \left(0,1\right)$, $p_{f}^{\left(l\right)}\in \left(0,1\right)$, T∈[0,5], and $T_{\mathrm{era}}\in \left[0,5\right]$. The optimization is performed with a maximum budget of 100 trials and terminates when the predefined trial budget is exhausted, after which the final optimized parameter set is obtained. As an example, for the product code based on $C_{1}\left[255,231,3\right]$, the optimized parameter set obtained by *Optuna* is given as follows:

$$\begin{array}{cc} w= & [3.4790,\;9.0176,\;4.2010,\;5.2347,\;7.8923,\;3.9892,\\ \; & 4.0141,\;5.7246,\;7.3568,\;5.9619,\;4.3605,\;4.8770], \end{array}$$

$$\begin{array}{cc} \beta = & [0.7302,\;0.4019,\;0.8285,\;0.4976,\;0.7970,\;0.6120,\\ \; & 0.6074,\;0.6438,\;0.8120,\;0.7579,\;0.9121,\;0.4449], \end{array}$$

$$\begin{array}{cc} p_{f}= & [0.5329,\;0.4829,\;0.0301,\;0.3982,\;0.9523,\;0.4490,\\ \; & 0.2613,\;0.1214,\;0.7795,\;0.2944,\;0.4397,\;0.1018]. \end{array}$$

with

$$T=0.9336,\;T_{\mathrm{era}}=1.2495.$$

### 5.2. The Influence of EaED

In this subsection, we investigate the impact of EaED on the performance of RC-iBDD-RF, as well as on the execution number of BDD. We select the BCH code $C_{1}\left[255,231,3\right]$ as the component code and employ the hyperparameter optimization tool *optuna* to obtain all parameters of RC-iBDD-RF. The number of iterations is set to 12.

**Figure 5:**
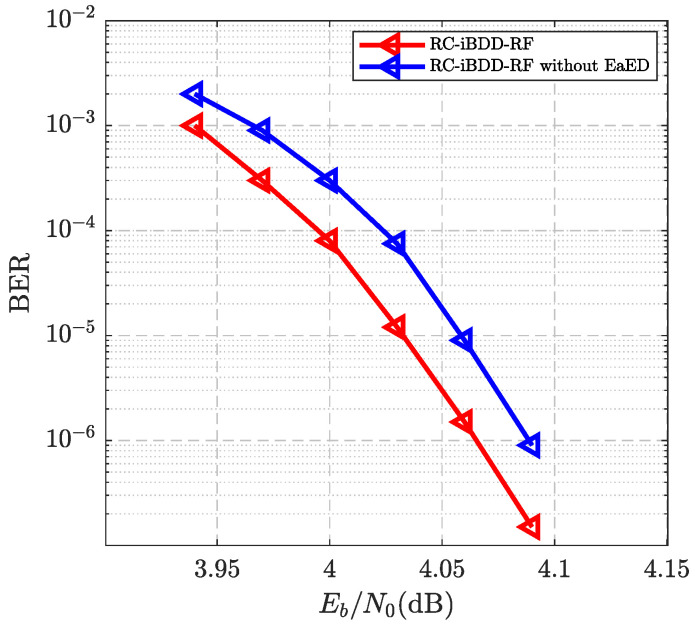
The performances of RC-iBDD-RF with and without EaED. The code $C_{1}$ is selected as the component code.

**Figure 6:**
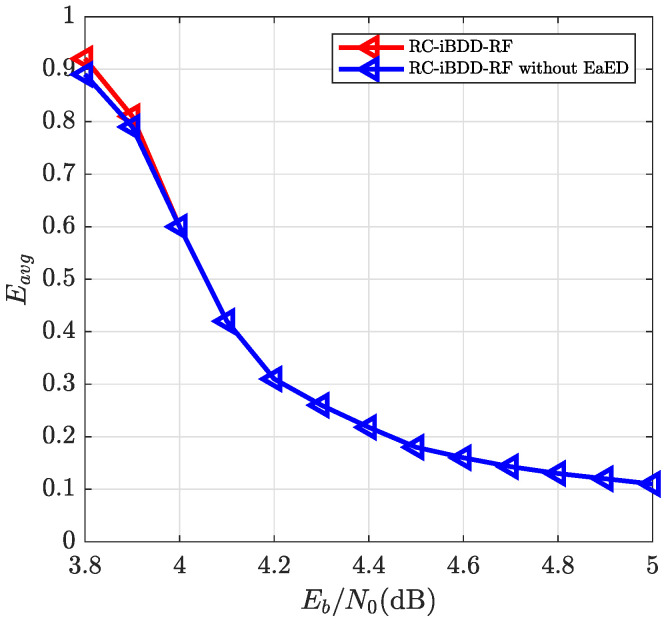
The average execution numbers of BDD for RC-iBDD-RF with and without EaED. The code $C_{1}$ is selected as the component code.

In Figure 5, we present the performance of RC-iBDD-RF with and without EaED. It can be seen that at $\mathrm{BER}=10^{-6}$, RC-iBDD-RF with EaED achieves an approximately 0.03 dB gain over RC-iBDD-RF without EaED. To further investigate its impact on complexity, we present simulation-based comparisons of the average execution numbers of BDD for the RC-iBDD-RF with and without EaED in the following. In Figure 6, we present $E_{\mathrm{avg}}$ for RC-iBDD-RF without EaED and RC-iBDD-RF with EaED. The BCH code $C_{1}\left[255,231,3\right]$ is selected as the component code. From Figure 6, we observe that RC-iBDD-RF without EaED and RC-iBDD-RF with EaED admit almost the same average execution numbers of BDD. This is due to the fact that, in RC-iBDD-RF, bit erasures are applied only once and are controlled by the threshold $T_{\mathrm{era}}$. These results indicate that RC-iBDD-RF with EaED has almost the same decoding complexity as the RC-iBDD-RF without EaED, while achieving superior performance.

We point out that the EaED post-processing contributes only a small additional gain, about 0.03 dB at BER $=10^{-6}$ in the considered simulation. Therefore, EaED should be regarded as an optional residual-error cleanup module rather than the main source of the coding gain. Since the fixed filling patterns are applied only once after the iterative decoding process, this module does not introduce extra global decoding iterations or random pattern search. Thus, it may be enabled in low-BER-oriented applications where a small deterministic post-processing cost is acceptable, while it can be disabled in systems with more stringent latency or complexity constraints.

### 5.3. Performance Comparison for RC-iBDD-RF

In this subsection, we present the performance of RC-iBDD-RF over the AWGN channel and compare it with several existing SA-HDDs, including iBDD-RF, iBDD-SR, the threshold-based binary messaging-passing decoder (TB-BMPD), and DRSD.

**Figure 7:**
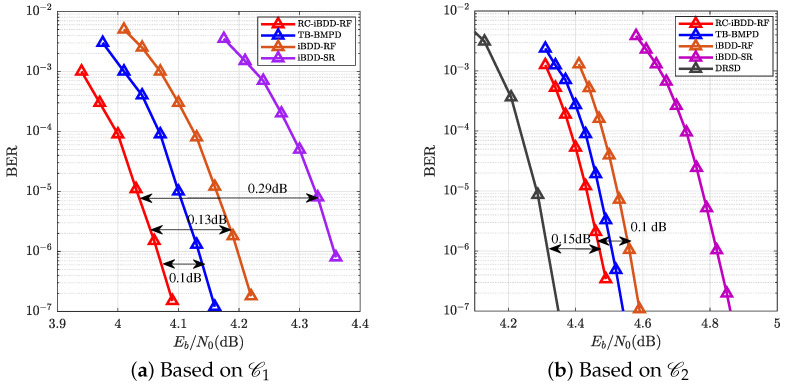
Performance comparison between RC-iBDD-RF and various SA-HDDs.

We select the representative high-rate BCH codes $C_{1}\left[255,231,3\right]$ and $C_{2}\left[256,239,2\right]$ [31,34,35] as component codes and set the number of iterations to 12. For TB-BMPD, in addition to ten algorithm iterations, two extra BDD iterations are required. For DRSD, only 10 iterations are performed, and the component codes are chosen as $C_{1}$ and $C_{3}\left[255,238,2\right]$. We point out that, since the code rates of $C_{2}$ and $C_{3}$ are very close, the difference can be neglected. The performances of various SA-HDDs are shown in Figure 7a,b. Based on these two figures, we have the following observations.

- For the PC based on $C_{1}$, RC-iBDD-RF outperforms the iBDD-SR, iBDD-RF, and TB-BMPD. Specifically, its performance is approximately 0.29 dB better when compared with iBDD-SR, 0.13 dB better when compared with iBDD-RF, and 0.1 dB better when compared with TB-BMPD.
- For the PC based on $C_{2}$, RC-iBDD-RF achieves about 0.1 dB performance gain over iBDD-RF, while it is only 0.15 dB away from DRSD. Note that it was shown in [30] that the decoding complexity of DRSD is higher than that of iBDD-RF. This indicates that the decoding complexity of RC-iBDD-RF is much lower than that of DRSD.

Within the simulated BER range in Figure 7, no obvious error floor is observed for RC-iBDD-RF. The low-BER gain suggests that the proposed reliability metric and the EaED-based post-processing can reduce part of the residual errors after iterative BDD. However, as with other iBDD-based decoders, rare decoding failures may still occur at very low BER due to stall patterns or miscorrection-induced residual patterns. The fixed filling patterns in EaED provide deterministic low-complexity post-processing and avoid random pattern search, but they may not cover all possible rare events in the deep error-floor region. A complete error-floor characterization would require further importance-sampling simulations or analytical stall-pattern analysis, which is an interesting topic for future research.

### 5.4. Complexity-Performance Trade-Off Analysis

To further provide a clearer and fairer comparison among different decoders, we introduce a complexity-storage-weighted SNR metric inspired by the latency-weighted decoding metric in [36]. In [36], the decoding performance is evaluated by jointly considering the error-rate performance and the normalized decoding delay. Since this work focuses on algorithm-level decoding complexity and memory storage rather than hardware-measured latency, we replace the normalized decoding delay with two normalized cost factors: normalized computational complexity and normalized storage complexity.

For decoder *d*, the normalized computational complexity is defined as

(18) $$C_{d}^{\mathrm{norm}}=\frac{C_{d}}{C_{\mathrm{iBDD}\text{-}RF}},$$

where $C_{d}$ denotes the computational complexity of decoder *d*, and $C_{\mathrm{iBDD}\text{-}RF}$ denotes the computational complexity of iBDD-RF. Similarly, the normalized storage complexity is defined as

(19) $$M_{d}^{\mathrm{norm}}=\frac{M_{d}}{M_{\mathrm{iBDD}\text{-}RF}},$$

where $M_{d}$ denotes the memory requirement of decoder *d*.

The normalized required SNR at a target ${\mathrm{BER}}_{0}$ is defined as

(20) $$\Gamma_{d}\left({\mathrm{BER}}_{0}\right)=\frac{10^{\gamma_{d}\left({\mathrm{BER}}_{0}\right)/10}}{10^{\gamma_{\mathrm{iBDD}\text{-}RF}\left({\mathrm{BER}}_{0}\right)/10}}=10^{(\gamma_{d}\left({\mathrm{BER}}_{0}\right)-\gamma_{\mathrm{iBDD}\text{-}RF}\left({\mathrm{BER}}_{0}\right))/10},$$

where

(21) $$\gamma_{d}\left({\mathrm{BER}}_{0}\right)={\left.\frac{E_{b}}{N_{0}}\right|}_{\mathrm{BER}={\mathrm{BER}}_{0}}$$

denotes the required $E_{b}/N_{0}$ for decoder *d* to achieve the target ${\mathrm{BER}}_{0}$.

The complexity-storage-weighted SNR is defined as

(22) $$\;\Omega_{d}\left({\mathrm{BER}}_{0}\right)=\Gamma_{d}\left({\mathrm{BER}}_{0}\right)\cdot C_{d}^{\mathrm{norm}}\cdot M_{d}^{\mathrm{norm}}.$$

A smaller $\Omega_{d}\left({\mathrm{BER}}_{0}\right)$ indicates a better trade-off between decoding performance, computational complexity, and memory storage.

For the product code based on $C_{1}\left[255,231,3\right]$, the memory requirements of RC-iBDD-RF and iBDD-RF are 65,097q+765 bits and 65,073q bits, respectively. Without loss of generality, we set q=5. The computational complexity of RC-iBDD-RF and iBDD-RF are $O\left(2.52n^{3}\right)+O\left(3.72n\right)$ and $O\left(5.832n^{3}\right)+O\left(3.86n\right)$, respectively. At the target BER ${\mathrm{BER}}_{0}=10^{-6}$, the required $E_{b}/N_{0}$ of RC-iBDD-RF and iBDD-RF are 4.06 dB and 4.20 dB, respectively, according to Figure 7a.

Accordingly, the complexity-storage-weighted SNR is calculated as

(23) $$\;\begin{array}{cc} \Omega_{\mathrm{RC}\text{-}\mathrm{iBDD}\text{-}\mathrm{RF}}\left(10^{-6}\right)\; & =\Gamma_{\mathrm{RC}\text{-}\mathrm{iBDD}\text{-}\mathrm{RF}}\cdot C_{\mathrm{RC}\text{-}\mathrm{iBDD}\text{-}\mathrm{RF}}^{\mathrm{norm}}\cdot M_{\mathrm{RC}\text{-}\mathrm{iBDD}\text{-}\mathrm{RF}}^{\mathrm{norm}}\\ \; & =0.4195. \end{array}$$

Since the metric is normalized to iBDD-RF, the corresponding value of iBDD-RF is 1. RC-iBDD-RF achieves a value smaller than 1, which indicates a better trade-off between decoding performance, computational complexity, and memory storage.

## 6. Conclusions

In this paper, we proposed the RC-iBDD-RF, a reduced-complexity random flipping decoder for product codes aided by erasure. We first presented an enhanced reliability metric. We then proposed to use EaED as a post-processing procedure to handle the residual errors. The complexity of the proposed decoder was investigated in terms of both memory storage and computational complexity. The analysis showed that the computational complexity of RC-iBDD-RF is lower than that of iBDD-RF. We also presented extensive numerical results to show the performance advantages of RC-iBDD-RF over iBDD-RF. Moreover, the proposed complexity-storage-weighted SNR metric further demonstrates that RC-iBDD-RF achieves a more favorable trade-off between decoding performance, computational complexity, and memory storage. These results confirm the potential of RC-iBDD-RF for high-speed communication systems.

## Figures and Tables

**Table 1:** Complexity comparison of RC-iBDD-RF, iBDD-RF, and iBDD.

Algorithm	Additional Memory Required	Computational Complexity
RC-iBDD-RF	$(6l_{\mathrm{RC}}+n^{2})q+3n$	$O\left(2l_{\mathrm{RC}}E_{\mathrm{avg}}n^{3}\right)+O\left(2l_{\mathrm{RC}}nN_{\mathrm{avg}}\right)$
iBDD-RF	$(4l_{\mathrm{RF}}+n^{2})q$	$O\left(2l_{\mathrm{RF}}E_{\mathrm{avg}}n^{3}\right)+O\left(2l_{\mathrm{RF}}nN_{\mathrm{avg}}\right)$
iBDD	0	$O(2l_{\mathrm{iBDD}}E_{\mathrm{avg}}n^{3})$

## Data Availability

The original contributions presented in this study are included in the article. Further inquiries can be directed to the corresponding author.
